# Sport development in rural schools of Lephalale in Limpopo province: Barriers and facilitators

**DOI:** 10.4102/sajp.v80i1.2004

**Published:** 2024-05-20

**Authors:** Tulycia M. Letshokotla, Douglas Maleka, Mary L. Galantino, Rethabile Nkuna

**Affiliations:** 1 Department of Physiotherapy, Faculty of Health Sciences, Sefako Makgatho Health Science University, Pretoria, South Africa; 2 Department of Physiotherapy, Faculty of Health Sciences, Stockton University, New Jersey, United States

**Keywords:** rural schools, sports development, physical activity, facilitators, barriers

## Abstract

**Background:**

Sports development and promotion of physical activities (PA) through various sports in rural schools of South Africa (SA) is essential to optimise growth and wellbeing of children. There is a paucity of research specific to rural areas, and this is implicated on the lack of resources, effective programmes as well as resources to promote structured PAs and sports.

**Objectives:**

To explore sports development facilitators and barriers in rural schools.

**Method:**

We conducted an exploratory qualitative study and recruited Life Orientation (LO) teachers and school principals. We established structured interview guidelines and recorded the interviews which were transcribed verbatim. Data saturation was reached by the eighth participant. The data were analysed using thematic content analysis.

**Results:**

Participating schools experienced shared challenges in developing and promoting PAs. Five themes emerged addressing the barriers: sport facilities, time management, workload, financial constraints, and lack of participation. Six categories emerged as facilitators: intrapersonal factors, interpersonal factors, personal, social, physical and mental benefits.

**Conclusion:**

Most rural schools in Lephalale district struggle to promote and develop sports because of several targeted factors. These schools have little to no strategic plans to develop and promote sports because of the prioritisation of the core curriculum and/or examinable subjects in classroom duties which is deemed their highest priority.

**Clinical Implication(s):**

Implementation of tailored sports development policies in rural schools via acquisition of resources, education regarding the positive impact of sport, and focused planning is required. Healthcare professionals such as physiotherapists may aid in the encouragement of sports.

## Introduction

In 2018, the esteemed World Health Organisation (WHO) launched a programme aimed at promoting physical activity (PA) throughout the global education sector (WHO 2018). This programme uncovered the many health benefits of consistent participation in sports and PAs, including a robust and functional musculoskeletal system, healthy body weight, positive psychological well-being, enhanced academic performance, and valuable social and economic advantages (WHO 2020).

The state of sports development in rural schools of South Africa is concerning, primarily because of the inadequate resources allocated for promoting PAs and sports (Mthombeni et al. 2023). Consequently, there has been a substantial increase in non-communicable diseases (NCDs) like diabetes, hypertension, obesity, cardiovascular diseases, and cancer (WHO 2020). It’s disheartening to note that a large majority of South African teenagers are physically inactive, contributing to the high rates of obesity among children and youth (Adom et al. 2019).

The absence of adequate sports infrastructure in numerous rural schools continues to pose a challenge, mainly because of the enduring consequences of apartheid (Mthombeni et al. 2023). Left unaddressed, this dearth of resources may result in the departure of extraordinarily gifted athletes and ultimately affect the competitiveness and overall calibre of the national sports teams (Kruger & Pienaar 2011).

This research is critical since there are few studies on the challenges of sports development in rural schools of Lephalale, Limpopo province. There is also limited literature on promoting PAs through sports in rural schools throughout the country. Therefore, the goal of this study is to explore the barriers and facilitators for PAs and sports development in rural schools of Lephalale, Limpopo province, South Africa. Identifying these challenges and opportunities will provide valuable insights to communities, teachers, students, and administrators, enabling them to minimise barriers and maximise opportunities for promoting PAs.

Promoting PA is critical in preventing NCDs in children. Implementing sustainable PAs can create a healthier future for the next generation. All stakeholders must work together to meet the standards for promoting PA and ensure that every child in South Africa has access to the benefits of sports and PAs.

## Research methods and design

### Study design

An exploratory qualitative study was conducted through in-depth interviews of Life Orientation (LO) teachers and school principals in rural Lephalale, Limpopo province, South Africa.

### Study setting

The research study was conducted purposively in schools in the Phalala North circuit in Lephalale, located in the northern region of Limpopo province, South Africa. This area is predominately a rural setting. The Phalala North circuit comprises a total of 31 schools, out of which 19 are primary and 12 are secondary schools.

### Study population and sampling strategy

The study selected schools from the Phalala North Circuit based on a list provided by the district Department of Education. This circuit comprises of non-fee-paying schools that primarily serve low socio-economic populations in the area. The study involved 14 schools, consisting of 9 primary and 5 secondary schools. During data collection, the schools provided the principal (headmaster) and LO teachers to participate in the study. Principals were selected based on their knowledge of the daily school operations and funds allocation, whereas LO teachers were selected on the basis of their subject which focuses on promoting a healthy lifestyle.

### Data collection

#### The instrument

The data collection instrument was a self-constructed interview schedule with guidelines including four sections. The introductory section consists of the participant’s demographic information such as age, gender, education level, and participants role in the school (LO teacher or school principal). The remaining four sections included: (1) information regarding the schools’ sports development challenges; (2) participants’ views on introducing new and various sporting codes in the schools; (3) information about strategic plans to implement the promotion of sports and PAs for students; and (4) assessed facilitators that could influence sports development and promote PAs in schools (see Appendix 1). To ensure the validity of the tool, a panel of experts that included the supervisor and two lecturers familiar with qualitative research were selected to oversee the process. A pilot study was conducted with two schools to ensure its reliability.

#### Procedure

From 10 June 2020 through 22 July 2020, the researcher held one-on-one interviews with each of the candidate. These interviews were conducted privately. At the beginning of every interview, the researcher explained the aims and objectives of the study. The opening question was about the different types of sporting activities. During the interview, the interviewer listened carefully to the participants’ answers and provided prompts when necessary. To keep a record of the interviews, the researcher took notes and used a voice recorder. Each interview took a minimum of 10–15 min and a maximum of 30 min. After collecting the data, the researcher observed the sports equipment and playing fields.

### Data analysis

All the interviews were recorded and transcribed verbatim for analysis. Data saturation was reached after the eighth participant. Inductive thematic content analysis was used which allows for a nuanced exploration of the data and it revealed unexpected insights. The researcher generated and coded data and produced themes to allow for the identification of themes and sub-themes emerging from the data to make sense of the data. The researcher familiarised herself with data by being immersed in raw data; read and re-read transcripts, and field notes. Coding was done by identifying and labelling interesting features, and patterns of data. Generation of potential themes was based on grouping codes that share similarities or connections. Potential themes are reviewed and refined, to iterate process, merging, splitting or discarding themes. This was followed by defining and naming themes and applying them to the entire data set. The researcher always engaged in constant comparison. This was followed by member checking to review and report the findings with reflexivity.

### Ethical considerations

The protocol of the study was approved by the host university’s research ethics committee (SMUREC/H/347/ 2018: PG). Permission to conduct the study at the schools of Lephalale was approved by the Department of Education in Limpopo province South Africa. During data collection, participants were aware that engagement in interviews was voluntary. The demographic information was deidentified and coded to assure confidentiality as guided by the research protocol. The researcher explained the purpose of the study and later informed the participants that they might withdraw from the study at any juncture without repercussions. All Life Orientation teachers and school principals signed the informed consent.

## Results

### Barriers

**FIGURE 1:**
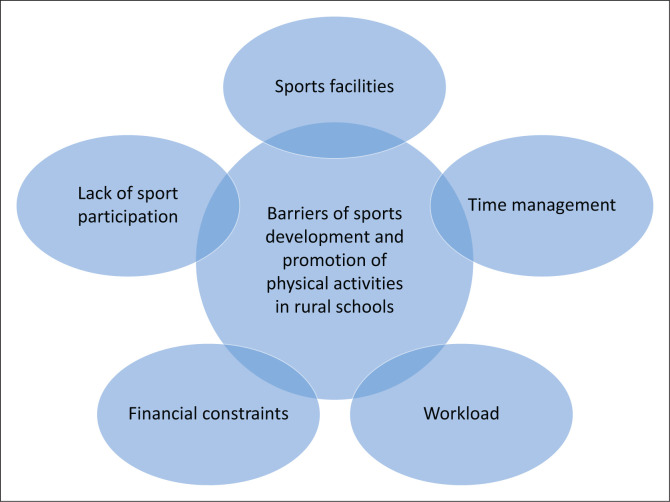
Illustration of barriers of sports development and promotion of physical activities in schools.

Five themes and sub-themes emerged as challenges of sport development in rural schools. Table 1 presents barriers of sports development and promotion of PAs in rural schools and that five themes and sub-themes emerged as indicated in Figure 1.

**TABLE 1:** Themes, sub-themes, and participants’ responses on matters relating to barriers on sports development challenges in the rural schools of Lephalale.

Number	Theme	Sub-theme	Quotations
01	Sports facilities	Infrastructure	‘[*T*]here aren’t any proper facilities to introduce new sports codes in the school. Because instead of having a proper netball court, the children play on the dusty soiled and rocky grounds and sometime is not conducive to play …’ (P4) ‘One of the issues that we are currently facing is the yard space. As you know that a soccer needs bigger space, the learners are unfortunately unable to play in the school premises, but rather we ask the local municipality to allow the children to use the community sports grounds–of which is a huge problem because the sports ground is not always available for use. And for that reason, we decided not to participate in soccer matches with other participating schools.’ (P1)
02	Time management	Individual time constraints	‘[*D*]because of time management challenges, we have decided to take sports only on Wednesday afternoon, as it is known as sports day. And sometimes they play alone without teachers or someone to coach them.’ (P3)
		Timetables	‘[*I*]f the Department of Education decides to put sports in the curriculum, it should fit in the timetable like other subjects, the department wants us to allow learners take part in sports but there isn’t any time for it, we just created it on our own and that is giving us a challenge …’ (P3)
03	Workload	Teachers’ workload	‘[*O*]ne other challenge that we have in the school is teachers’ workload, our work is too much …’ (P7)
		Learners’ workload	‘Honestly speaking, sometimes it is difficult for some learners to take part in sports because sometimes you would find that schoolwork is too much.’ (P5)
04	Financial constraints	Government funds	‘We need money from the government to help us implement sports in the school …’ (P4)
		School budgets	‘Even if we think about introducing new sport codes like tennis and swimming in the school, the truth is that the school cannot afford, because you’ll find that the budget allocated for sports is only 10%…while the other funds are for academics and feeding scheme in the school …’ (P3)
05	Lack of Participation	Learners’ interests in sports	‘Most of our female learners are not participating in sports as compared to male learners …’ (P1) ‘The school does not have coaches and resources to implement some commonly known sports in our community such as netball and soccer. We have noticed that it has affected the learners’ interest in sports…they drop out of sports teams …’ (P3)
		Sports tournaments	‘We entered a chess competition last year with three learners competing. Only two learners managed to succeed in that competition, but those leaners couldn’t go any further provincially and nationally since the competition was only held locally. This year we haven’t had competitions because most schools locally do not play chess, but our learners are playing among themselves…and when it comes to other sports like volleyball, netball and soccer we only have friendly games with other schools because we hardly compete in those sport codes… the only time when our learners had ever reached a district level in competitions was one time when two learners succeeded in athletics [*100 m*].’ (P2)

### Facilitators

**FIGURE 2:**
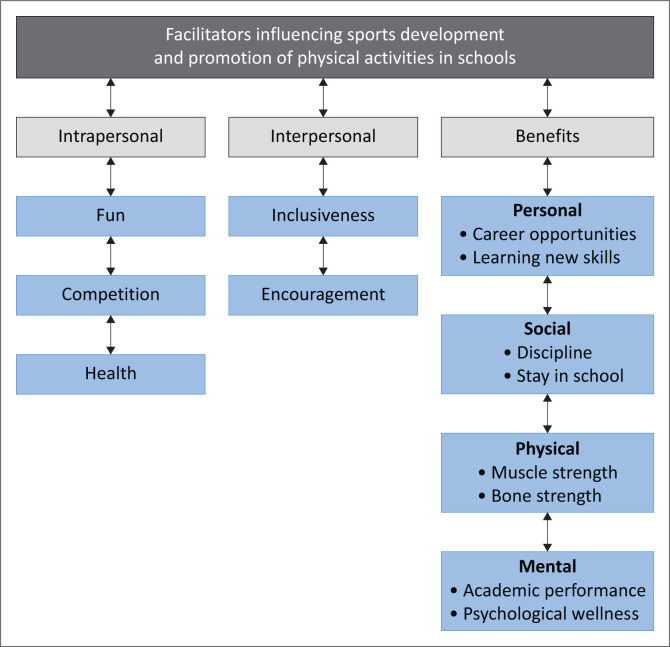
Facilitators that influenced sports development and promotion of physical activities.

Six categories emerged regarding **facilitators** that influenced sports development and promotion of PA in rural schools. The participants gave their views and opinions on how more and different sports codes can enhance extracurricular activities. They provided perspectives on how teaching physical education as a school subject in the classroom can promote PA and sports among the learners (see Figure 2, Table 2 and Table 3).

**TABLE 2:** Categories, sub-categories, and participants responses which illuminate the importance of adding sport codes during extracurricular activities.

Number	Categories	Sub-categories	Quotations
01	Intrapersonal factors	Fun	‘[*T*]he learners would be so excited about it and would actually have fun …’ (P2) ‘I believe the learners would enjoy it …’ (P5)
		Health	‘[*C*]hildren would benefit greatly, health wise …’ (P6) ‘Sport is known to keep individuals healthy and active …’ (P2)
		Competition	‘[*I*]t would definitely increase the children’s competitive spirit…we were so proud of our soccer boys because they always bring in good results from competition games …’ (P7) ‘We normally compete for friendly matches. But now we have one learner who won the netball competition for the circuit, and she is supposed to represent us at a provincial level in Polokwane this week …’ (P3)
02	Interpersonal factors	Inclusiveness	‘It’s a good idea because some subjects like LO already encourage PA …’ (P8) ‘[*S*]ome learners are really interested in soccer or netball, so I have noticed that they do not even bother participating, so maybe if we can introduce other sport codes, they may consider taking part …’ (P4)
		Encouragement	‘It would be good because some learners are not intellectually gifted, as some are underperforming, so the teaching of PA might encourage more pupils to take part in sports.’ (P1) ‘If they take the extracurricular activities serious, some learners might thrive in that field.’ (P3) ‘[I]t would be good; this would encourage some learners to participate in sports and some form of PA …’ (P3)

LO, Life Orientation; PA, physical activity.

**TABLE 3:** Categories, sub-categories, and participants quotations on views and opinions regarding teaching sports-related subjects in classrooms.

Number	Categories	Sub-categories	Quotations
01	Personal benefits	Career opportunities	‘[*T*]hrough knowledge and experience they would be getting in the physical education class, some learners who are currently excelling in sports now would consider pursuing sports as a career.’ (P8)
		Learning new skills	‘[*I*]t is good…skills and talents would be nurtured …’ (P2)
02	Social benefits	Discipline	‘What I have noticed is that most kids who are part of a team or let’s just say taking part in some form of sports, those kids are very disciplined as compared to others.’ (P1)
		Stay in school	‘The teaching of physical education in the classroom would help bring many learners who had dropped out to come back to school.’ (P7) ‘[*E*]ven when it is after school, they can still play to keep them off the streets …’ (P3)
03	Physical fitness	Strong muscles	‘[*I*]t’s good… It would encourage them to be physically fit, therefore improving muscle and bone strength …’ (P3)
		Strong bones	‘[*I*]t’s good… It would encourage them to be physically fit, therefore improving muscle and bone strength …’ (P3)
04	Mental wellness	Academic performance	‘[*A*]s we know sport has the ability to train the minds of learners and so this helps with academic performance and reliving stress related to their school workload.’ (P3)
		Psychological wellness	‘[*A*]s we know sport has the ability to train the minds of learners and so this helps with academic performance and reliving stress related to their school workload.’ (P8) ‘I read somewhere that sports is good for mental wellness.’ (P3)

## Discussion

### Barriers

During the study, participants highlighted various obstacles that hinder sports development in their schools. These barriers comprise insufficient sports facilities, ineffective time management, overwhelming academic workload, limited financial resources, and low student participation in sports.

#### Sports facilities

The study found that most schools participating in the research did not have the necessary sports facilities and infrastructure. Walter (2011) also reported that disadvantaged schools in rural areas often lack essential sports and recreation facilities. Similarly, Nongogo, Kubayi and Amusa (2014) noted that historically black-dominated communities had limited sports and recreational opportunities, a common issue in South African public schools. Walter (2011) emphasised that schools should be the primary site for enabling children to meet the World Health Organization’s PA recommendations, but it seems challenging as many schools’ face obstacles such as improper infrastructure.

According to Kubayi, Nongogo and Amusa (2014), public schools funded by the government and located in rural areas lack quality sports facilities. This is because of the inability of public schools to raise sufficient funds to construct and maintain the available sporting facilities. Some participants mentioned that their schools had problems with yard spacing, which hindered the proper development of sports grounds. They also faced difficulties proposing indoor sports activities and hosting tournaments on account of the lack of halls in their schools. According to Macupe (2018), rural schools in South Africa provide unsatisfactory services because of a lack of basic infrastructure and financial difficulties. Mchunu and Le Roux (2010) suggest that insufficient sports facilities in schools are a crucial factor that discourages adolescent students from participating in sports.

#### Time management

During the study, some participants discussed the challenge of maintaining academic and sports activities balanced throughout the year. They mentioned that some learners find it burdensome to manage their time during athletic seasons, which affects their academic performance. According to Owen (2016), time management skills are crucial for teachers and learners in schools. Additionally, Owen (2016) mentioned that there are no significant differences between the grades and overall stress levels of student-athletes who attend time management workshops.

Furthermore, some participants in the study reported that they were struggling to promote sports and PAs because of limited time as teaching and learning schedules should be given the biggest portion of time on the curriculum. With the literacy standards deteriorating, the teachers being judged by the employer based on examinable subjects, extracurricular activities such as sports are to be compromised. Eime et al. (2008) found that a lack of time management between academics and extracurricular activities is a common barrier to sports and PA participation. The British Council (2016) also highlighted the time-consuming nature of running school sports from a logical and administrative perspective, which many teachers acknowledged.

#### Workload

The participants of the study expressed that their workload and the nature of their work hinder them from promoting PA, even though they understand the significance of encouraging learners to engage in PA. Some participants reported that public schools do not have a balanced teacher-to-learner ratio, and teachers are burdened with the responsibility of teaching and managing overpopulated classrooms. Policymakers have mandated that teachers should integrate extra-curricular activities into their teaching to meet the integrated quality management system (IQMS) performance standards as this helps provide students with a well-rounded education and improves performance. Extracurricular activities help develop important life skills such as teamwork, leadership, time management, and communication and can lead to discovering interests and passions. The challenge which merges from this is that some unions have argued that teachers should be compensated for any time spent outside of the compulsory 1800 hours (Fedsas 2012).

#### Financial constraints

According to this current study, participants have shown concern regarding the inadequacy of government funding for sports programmes in schools. The participants indicated that funds are mainly focused on academic improvement and feeding schemes, leaving insufficient resources for sports. The study’s findings have confirmed that schools with low socio-economic backgrounds struggle to support sports activities because of limited funding (Elumilade et al. 2006).

Furthermore, the participants in the study indicated that many schools cannot introduce new sporting codes such as swimming and cricket, because of their limited annual budgets. Bisschoff and Koebe (2005) explained that each school under the provincial Department of Education (DoE) administration receives a specific amount of money at the beginning of each academic year. This funding is deposited directly into the Section 21 schools’ banking account, and should include resources for sports and recreation.

#### Lack of participation in sport

The present study found that the development of sports in schools is a challenging task as most students, especially female learners, exhibit less interest in sports as compared to their male counterparts. According to Beech et al. (2003), young girls tend to stop participating in PAs when they enter adolescence.

The participants in this study discovered that students, particularly in secondary schools, tend to become less interested in PAs and sports as they progress through school. This finding is supported by Cozett, Bassett and Leach (2016) who noted that South African adolescents are physically inactive and have concerning rates of obesity. It is crucial to encourage sports participation during this stage since adopting positive health behaviours provides an excellent opportunity for intervention (Mackintosh et al. 2011).

### Facilitators

The study participants, unequivocally, identified a range of intrapersonal and interpersonal factors, as well as a host of personal, social, physical and mental benefits that are instrumental in facilitating sports development and promoting PA in rural schools. Below, we elaborate on each point.

#### Intrapersonal factors

The study participants stated that introducing various sports programmes in schools creates excitement among learners to play, enjoy and learn through fun games. This aligns with the recommendations of WHO (2020), emphasising the importance of physical education in schools for sustained general health and well-being. World Health Organization (2020) recommends that physical education programmes should offer activities that enhance current health while teaching knowledge and skills that foster long-term commitment to PAs as part of a healthy lifestyle. The study also revealed that the participants have some knowledge about how frequent engagement in sports activities can benefit students’ health. Moreover, the participants of the study mentioned that including other sports codes in schools may encourage children to develop a competitive spirit and value teamwork. The findings of this study support Kanan and Al-Karasneh’s (2009) argument that children who participate in sports teams learn to appreciate the importance of teamwork as they play alongside their peers.

#### Interpersonal factors

Soccer and netball are the predominant sports in most rural schools, as stated by Kubayi et al. (2014). However, this concentration on only a few sports may have negative consequences, such as the exclusion of some students, discouragement and diversion from other activities (Sport New Zealand 2015). The participants of a study have highlighted the importance of introducing a variety of sports in rural schools, which would promote inclusivity among learners. Furthermore, the study’s participants emphasised that the introduction of diverse sporting codes in rural schools would encourage more students to participate in PAs. This would benefit those students who are not academically gifted, as sports can help them stay motivated and engaged in school (Taliaferro et al. 2011).

#### Personal benefits

The study’s participants expressed that physical education holds significant importance in the classroom as it can help shape and encourage skills and talents that may potentially lead to career opportunities in the future. This viewpoint agrees with Kirui, Kipkoech and Simotwo (2013)’s assertion that physical education is crucial for skills and talent development apart from academics. Evidence suggests that sports assist in maintaining balance in society by requiring individuals to work together towards setting and achieving goals (Kirui et al. 2013).

#### Social benefits

According to this study, the participants strongly believe that learners who participate in sports exhibit more discipline than those who do not engage in PA. Holt et al. (2011) found that sports participation provides social benefits such as positive relationships with coaches, making new friends, discipline and developing teamwork and social skills. This aligns with the findings of Howie et al. (2010), who suggest that children who participate in sports have better social skills than those who don’t engage in any PAs.

The participants of the study revealed that introducing physical education in schools could encourage students to stay in school and reduce their likelihood of being on the streets. While participating in sports and PA has positive social benefits, some studies suggest that negative peer pressure can affect the competitive nature of sports activities (Hansen, Larson & Dworkin 2003). However, another study before this one found that playing sports is associated with improved health outcomes, including a lower risk of emotional distress, substance abuse and crime (Taliaferro et al. 2011). In line with this, participants in the study mentioned how physical education as a school subject could help address behavioural issues among children and adolescents, such as wandering around the streets and contributing to criminal activities.

#### Physical fitness

The results of the study show that the participants know that regular exercise has a positive impact on strength and muscle development. Similarly, Malm et al. (2019) have demonstrated that engaging in PA or sports is essential for achieving physical fitness.

#### Mental wellness

Participants in the study have expressed awareness of the benefits of regular PA which can lead to improved academic performance, reduced stress levels, and enhanced brain function. This aligns with the findings of Cocke (2002). Furthermore, the participants reported knowledge of the positive effects of psychological wellness that can be improved through regular exercise. Taliaferro et al. (2011) supported this claim by stating that engaging in sports activities can reduce the likelihood of suicidal thoughts and intentions in comparison to those who don’t participate in any PA.

## Conclusion

The study’s findings paint a clear picture of the challenges regarding the development of sporting activities and promotion of PA faced by rural schools in the Lephalale district. Sports facilities, time management, workload, financial constraints and lack of sports participation are crucial barriers that these schools experience. However, the study also highlights some facilitators that can help overcome these challenges, such as fostering intrapersonal and interpersonal factors. Additionally, the participants’ extensive knowledge of the benefits of regular PA among students, which include: personal, social, physical, and mental benefits, underscores the importance of addressing these barriers and promoting PA in schools. With concerted efforts and the facilitation of these factors, these schools can overcome challenges and promote a healthy and active lifestyle among their students.
